# *In vivo* chemotherapeutic insight of a novel isocoumarin (3-hexyl-5,7-dimethoxy-isochromen-1-one): Genotoxicity, cell death induction, leukometry and phagocytic evaluation

**DOI:** 10.1590/1678-4685-GMB-2016-0316

**Published:** 2017

**Authors:** Flávio Henrique Souza de Araújo, Débora Rojas de Figueiredo, Sarah Alves Auharek, João Renato Pesarini, Alisson Meza, Roberto da Silva Gomes, Antônio Carlos Duenhas Monreal, Andréia Conceição Milan Brochado Antoniolli-Silva, Dênis Pires de Lima, Candida Aparecida Leite Kassuya, Adilson Beatriz, Rodrigo Juliano Oliveira

**Affiliations:** 1 Centro de Estudos em Célula Tronco, Terapia Celular e Genética Toxicológica, Hospital Universitário “Maria Aparecida Pedrossian”, Empresa Brasileira de Serviços Hospitalares, Campo Grande, MS, Brazil; 2 Programa de Mestrado em Farmácia, Centro de Ciências Biológicas e da Saúde, Universidade Federal de Mato Grosso do Sul, Campo Grande, MS, Brazil; 3 Faculdade de Medicina do Mucuri, Universidade Federal dos Vales do Jequitinhonha e Mucuri, Minas Gerais, MG, Brazil; 4 Programa de Pós-graduação em Saúde e Desenvolvimento na Região Centro-Oeste, Faculdade de Medicina “Dr. Hélio Mandetta”, Universidade Federal de Mato Grosso do Sul, Campo Grande, MS, Brazil; 5 Instituto de Química, Universidade Federal de Mato Grosso do Sul, Campo Grande, MS, Brazil; 6 Faculdade de Ciências Exatas e Tecnologias, Universidade Federal da Grande Dourados, Dourados, MS, Brazil; 7 Faculdade de Ciências da Saúde, Universidade Federal da Grande Dourados, Dourados, MS, Brazil; 8 Programa de Pós-graduação em Genética e Biologia Molecular, Centro de Ciências Biológicas, Universidade Estadual de Londrina, Londrina, PR, Brazil

**Keywords:** isocoumarin synthesis, genotoxicity, splenic phagocytosis, chemotherapy

## Abstract

Chemotherapy is one of the major approaches for the treatment of cancer. Therefore, the development of new chemotherapy drugs is an important aspect of medicinal chemistry. Chemotherapeutic agents include isocoumarins, which are privileged structures with potential antitumoral activity. Herein, a new 3-substituted isocoumarin was synthesized from 2-iodo-3,5-dimethoxy-benzoic acid and oct-1-yne in a cross-coupling Sonogashira reaction followed by a copper iodide-catalyzed intramolecular cyclization as key step using MeOH/Et_3_N as the solvent system. The present study also evaluated the leukometry, phagocytic activity, genotoxic potential and cell death induction of three different doses (5 mg/kg, 10 mg/kg and 20 mg/kg) of this newly synthesized isocoumarin, alone and in combination with the commercial chemotherapeutic agents cyclophosphamide (100 mg/kg) and cisplatin (6 mg/kg) in male Swiss mice. The results suggest that the isocoumarin has genotoxicity and causes cell death. Noteworthy, this new compound can increase splenic phagocytosis and lymphocyte frequency, which are related to immunomodulatory activity. When combined with either cyclophosphamide or cisplatin, chemopreventive activity led to a reduction in the effects of both chemotherapeutic drugs. Thus, the new isocoumarin is not a candidate for chemotherapeutic adjuvant in treatments using cyclophosphamide or cisplatin. Nevertheless, the compound itself is an important prototype for the development of new antitumor drugs.

## Introduction

The search for phenolic products with the potential to treat or cure cancer and/or to potentiate the cytotoxic effects of chemotherapeutic agents without having adverse effects on non-tumor cells is extremely important for the development of new chemotherapy drugs.

According to Pal *et al.* (2011) isocoumarins are characterized by a six membered heterocycle containing an oxygen atom in an unsaturated lactone, and represent an important group of natural products. They are useful intermediates in the synthesis of several hetero-carbocyclic compounds, including aromatic lactones called coumarins, which have an inverted lactone ring.

This class of compounds is known to have anti-inflammatory, antioxidant (Paya *et al.*, 1994; Murakami *et al.*, 2000; Agata *et al.*, 2004), apoptotic and anticarcinogenic potential (Paya *et al.*, 1994; Agata *et al.*, 2004; Chen *et al.*, 2007).

Recent studies have shown that isocoumarins induced the formation of reactive oxygen species (ROS), activated p53 signaling and enhanced apoptosis (Yin *et al.*, 2001). These characteristics are of interest for potential pharmaceutical applications (Yin *et al.*, 2001; Agata *et al.*, 2004; Chen *et al.*, 2007).

Several compounds can prevent cancer *via* their antigenotoxic action, or even treat tumors by causing extensive DNA damage, leading to apoptosis (Liu *et al.*, 2001; Fedel-Miyasato *et al.*, 2014; Mauro *et al.*, 2014). Thus, genotoxic and cell death assays, and tests that assess splenic phagocytosis and leukometry are widely used for initial assessment in the development of new drugs (Navarro *et al.*, 2014; Carvalho *et al.*, 2015). The present study describes the synthesis of a new isocoumarin, its toxicogenetic and cell death potential and its capability to alter splenic phagocytosis and leukometry alone and in combination with the chemotherapeutic agent cyclophosphamide and cisplatin in male Swiss mice.

## Material and Methods

### Chemistry

All reagents and spectrograde solvents for synthesis and nuclear magnetic resonance (NMR) measurements were purchased commercially and used without further purification.

#### NMR measurements

^1^H, ^13^C NMR and DEPT-135 spectra were recorded at room temperature on a Bruker 300 spectrometer (Institute of Chemistry, UFMS, Campo Grande, MS, Brazil) (10% in CDCl_3_ solutions at 298K) operating at 300.132 and 75.476 MHz, respectively. Data processing was carried out on a Solaris workstation. The ^1^H and ^13^C chemical shifts are given on the δ scale (ppm) and were referenced to internal tetra-methyl-silane (TMS); coupling constants (*J*) are reported in hertz (Hz). The abbreviations s, d, m and quint were used for simplet, douplet, multiplet and quintet, respectively.

#### Synthesis of compounds

**Figure 1:**
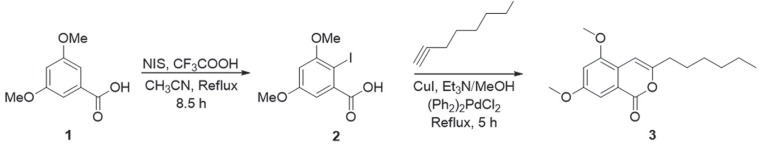
Synthesis of new isocoumarin (3) from precursor compounds 1 and 2.

2-iodo-3,5-dimethoxybenzoic acid (compound 2 in Figure 1): A mixture of 3,5-dimethoxybenzoic acid (200 mg, 1.0 mmol) and *N*-iodosuccinimide (NIS) (230 mg, 1.0 mmol) in the presence of CF_3_CO_2_H (0.025 mL, 0.3 mmol) in acetonitrile (10 mL) was stirred under reflux for 8.5 h. After extraction with AcOEt (315 mL), the combined organic layers were washed with distilled water (315 mL) and brine (315 mL), and dried over Na_2_SO_4_. The solvent was evaporated under reduced pressure and the product was purified by chromatographic column (hexane/AcOEt, 7:3) to achieve the pure compound in 79% yield.

^1^H NMR (300 MHz, CDCl_3_): δ (ppm) 6.98 (d, *J* = 2.7 Hz, 1H), 6.55 (d, *J* = 2.7 Hz, 1H), 3.87 (s, 3H), 3.83 (s, 3H).

3-hexyl-5,7-dimethoxy-isochromen-1-one (compound 3 in Figure 1): CuI (13.50 mg, 0.07 mmol) and (Ph_3_P)_2_PdCl_2_ (25.20 mg, 0.07 mmol) were added to a solution containing compound 2 (57.9 mg, 0.18 mmol) in 5.1 mL of trimethylamine and 5.0 mL of ethanol (freshly distilled and treated overnight with KOH and CaH_2_) and stirred for 30 min. Subsequently, 1-octyne (0.03 mL, 0.20 mmol) was added and the reaction mixture was stirred at 90 °C under nitrogen atmosphere and reflux was kept for 5 h. Then, the mixture was cooled until room temperature, extracted with hexane/acetate (7:3) (315 mL) and filtrated over Celite 535. The combined organic layers were washed with distilled water (315 mL), and dried over Na_2_SO_4_. Then, the organic solvent was evaporated under reduced pressure, to afford a brown solid in 90% yield without further purification steps.

^1^H NMR (300 MHz, CDCl_3_): δ (ppm) 0.86 (t, *J* = 6.65 Hz, 3H), 1.30 (m, 2H), 1.34 (quint, *J* = 6.64 Hz, 2H), 1.65 (m, 2H), 1.67 (quint, *J* = 6.72 Hz, 2H), 2.49 (t, *J* = 7.53 Hz, 2H), 3.86 (s, 3H), 3.87 (s, 3H), 6.52 (s, 1H), 6.79 (d, *J* = 2.34 Hz, 1H), 7.22 (d, *J* = 2.34 Hz, 1H). ^13^C NMR (75 MHz, CDCl_3_): δ (ppm) 14.04 (CH_3_), 22.50 (CH_2_), 27.02 (CH_2_), 28.67 (CH_2_), 33.52 (CH_2_), 55.79 (CH_3_), 55.87 (CH_3_), 97.07 (CH), 100.68 (CH), 105.09 (CH), 121.39 (C), 122.83 (C), 155.00 (C), 155.68 (C), 159.58 (C), 163.32 (C=O). DEPT-135 (↑) CH and CH_3_: 14.04, 55.79, 97.07, 100.65, 105.09; (↓) CH_2_: 22.50, 27.02, 28.67, 31.51, 33.52. GC-MS *m/z* (rel intensity) (EI, 70 eV): [M^+^] 290 (71.0%), 281 (28.8%), 253 (8.8%), 220 (22.2%), 219 (100.0%), 191 (37.7%), 178 (11.1%), 161 (13.1%), 149 (20.0%), 133 (17.7%), 84 (13.3%), 73 (22.2%), 57 (15.5%), 49 (17.7%).

#### Chemical agents, animals and experimental design

Two positive controls were used in the present study: cyclophosphamide and cisplatin. Cyclophosphamide (Fosfaseron^®^, Ithaca Laboratories, REG MS No. 1.2603.0056.002-1; Batch 063 020, Brazil) acts by alkylating cellular constituents through an indirect action that leads to the crosslinking of the DNA and to the disruption of transcription and translation. Cisplatin (Laboratory Intas Pharmaceuticals LTD, REG MS1.5537.0002.003-7; Matoda 382210, India) is an antineoplastic and antitumor agent that binds to DNA, resulting in intercalating links that induce structural changes and promote DNA transcription and replication inhibition. The chemotherapeutic agent cyclophosphamide was prepared in saline solution (pH 7.4) and administered at a concentration of 100 mg/kg (Oliveira *et al.*, 2009a), and cisplatin was administered at 6 mg/kg body weight (bw) (Oliveira *et al.*, 2009b). Both agents were administered intraperitoneally (ip) in a single dose. The new isocoumarin was first diluted in dimethylsulfoxide (DMSO) (1% final concentration) and then in Milli-Q water and administered at doses of 5, 10, and 20 mg/kg (bw, ip) (Chen *et al.*, 2007).

One hundred and twenty sexually mature Swiss strain (*Mus musculus*) male mice were obtained from the Central Animal Laboratory of Biological Sciences Center and Health, Federal University of Mato Grosso do Sul (CCBS/UFMS) and divided into 12 experimental groups (n = 10 animals per group). The animals were housed in polypropylene cages covered with wood shavings and maintained with a commercial diet (Nuvital^®^) and filtered water *ad libitum*. A 12 h photoperiod (12 h light, 12 h dark) was used, and the temperature and humidity were set at 22±2 °C and 55±10%, respectively, in a ventilated rack (ALESCO^®^, Brazil). The experiment was conducted as described in the rules of the Brazilian College of Animal Experimentation and according to the guidelines of the Universal Declaration of Animal Rights, with the approval of the Ethics Committee on Animal Experiments of UFMS under protocol 399/2012.

The 12 experimental groups were divided as follows:

Negative control group: The animals received the vehicle of isocoumarin (1% DMSO) and of cyclophosphamide (saline – 0.9% NaCl solution) at a dose of 0.1 mL/10 g bw, ip simultaneously.

Cyclophosphamide group: The animals received isocoumarin vehicle (0.1 mL/10 g bw) and cyclophosphamide (100 mg/kg bw, ip) simultaneously.

Cisplatin group: The animals received isocoumarin vehicle (0.1 mL/10 g bw) and cisplatin (6 mg/kg bw, ip) simultaneously.

Isocoumarin group: The animals received isocoumarin at three different doses, *i.e.*, 5 mg/kg, 10 mg/kg and 20 mg/kg bw, ip and cyclophosphamide vehicle (0.1 mL/10 g bw, ip), simultaneously.

Combination Group I (isocoumarin + cyclophosphamide): The animals received the isocoumarin in three different doses, *i.e.*, 5 mg/kg, 10 mg/kg and 20 mg/kg bw, ip, and cyclophosphamide (100 mg/kg bw, ip), simultaneously.

Combination Group II (isocoumarin + cisplatin): The animals received the isocoumarin in three different doses, *i.e.*, 5 mg/kg, 10 mg/kg and 20 mg/kg bw, ip, and cisplatin (6 mg/kg bw, ip), simultaneously.

Peripheral blood samples (20 μL) were collected for the comet and micronucleus test at 24 hours (T1) and for the micronucleus test at 48 (T2), and 72 hours (T3) after administration of the compounds. At T3, 20 μL peripheral blood was also collected for differential blood cell analysis. Seventy-two hours after application of the test compounds, the animals were sacrificed by cervical dislocation for organ collection.

### Biological assays

#### Peripheral blood micronucleus assay

The peripheral blood micronucleus assay was performed according to Hayashi *et al.* (1990) with modifications by Oliveira *et al.* (2009a). A drop of peripheral blood was deposited on a slide previously coated with 20 μL of acridine orange (1.0 mg/mL), and the slide was covered with a coverslip. The slide remained in a freezer (–20 °C) for two weeks. Analysis was performed using a fluorescence microscope (Bioval^®^, L Model 2000A) under 400x magnification with a 420–490 nm excitation filter and a 520 nm barrier filter.

#### Cell death assay

For the cell death assay, 100 μL of a solution of macerated liver or kidney was smeared onto a slide. Then, the slide was fixed in Carnoy solution for 10 min. The slide was subjected to decreasing concentrations of ethanol (95%–25%) and was then soaked in McIlvaine buffer for 10 min, followed by staining with acridine orange (0.01%, 5 min) and a final wash with McIlvaine buffer for 10 min. The identification of dead cells was performed by analyzing DNA fragmentation patterns according to Rovozzo and Burke (1973) and Mauro *et al.* (2011).

#### Splenic phagocytosis assay

The splenic phagocytosis assay was performed by homogenizing the spleen in physiological solution, resulting in a cell suspension. A 100 μL aliquot of the cell suspension was placed on a slide and covered with a coverslip. Before applying the cell suspension, 20 μL of acridine orange (1.0 mg/mL) had been added to cover the entire surface of each preheated slide. The analysis was performed using a fluorescence microscope (Bioval^®^, Model 2000A L) at a magnification of 400 with a 420–490 nm filter and a 520 nm barrier filter (Ishii *et al.*, 2011). In total, 100 cells per animal or 1000 cells per group were analyzed. The analysis of cells for the presence or absence of phagocytosis was based on the description of Hayashi *et al.* (1990).

#### Differential blood cell count

To perform the differential blood cell count, 20 μL of peripheral blood was smeared onto each histological slide. These were dried in open air and stained with Giemsa (10%) for 10 min. The slides were analyzed under bright field microscopy at 1000 magnification. In total, 100 cells/animal were analyzed and classified as lymphocytes, neutrophils, monocytes, eosinophils or basophils (Ishii *et al.*, 2011).

#### Calculation of percent damage reduction (DR%)

The calculation of the DR% was performed to evaluate the chemopreventive properties of the novel compound when combined with a genotoxic substance according to Manoharan and Banerjee (1985) and to Waters *et al.* (1990). The DR% was calculated as follows: [the mean of the positive control – the mean of the combination treatment] / (the mean of the positive control – the mean of the negative control). The result was multiplied by 100 to obtain the DR%.

### Statistical analysis

Data were analyzed by ANOVA with Tukey's post-hoc test using GraphPad Prism software (version 2.3; Graph-Pad Software Inc., San Diego, CA, USA). The values are reported as the mean±SE. The significance level was set at p < 0.05.

## Results

### Synthesis

As shown in Figure 1, the synthesis of isocoumarin (compound 3) was performed in good yields starting from commercially available 3,5-dimethoxybenzoic acid (compound 1). In the first step, the electrophilic aromatic iodination of compound 1 was performed using NIS in acetonitrile in the presence of a catalytic amount of trifluoroacetic acid. The corresponding iodinated compound 2 was obtained in 79% yield. Following, a cross-coupling Sonogashira reaction was carried out between the halogenated compound 2 and 1-octyne in a NEt_3_/MeOH solvent system to obtain the respective 2-alkynyl benzoic acid formed *in situ* which was treated with NEt_3_/MeOH to form the respective ammonium salt. This salt underwent CuI-catalyzed cyclization to give isocoumarin (3) in 90% yield.

The reason for this regioselectivity to form the isocoumarin (3) associated with a catalytic system using CuI and (Ph_3_P)_2_PdCl_2_ is not yet fully elucidated in the literature. According to Subramanian *et al.* (2005), the direction of this cyclization can be influenced by the nature of the catalyst systems, as well as by the solvent used in these reactions.

### Biological assays

#### Biometric parameters

The biometric parameter analysis showed no difference between the mean values of initial and final body weights of the analyzed mice (Table 1). The absolute organ weights are presented in Table 2. Significant increases in lung and spleen weights were observed in the groups treated with single doses of isocoumarin (5, 10 and 20 mg/kg). Increases in lung and spleen weights were also observed in the groups treated with isocoumarin combined with either chemotherapeutic agent (p < 0.05), although these increases were more prominent in the group that received isocoumarin alone.

**Table 1:** Means±SE of the initial and final weights of Swiss mice treated with different doses of isocoumarin.

Experimental groups	Initial weight (g)	Final weight (g)
Negative control	35.8±0.66 ^a^	35.0±0.75 ^a^
Isocoum 5 mg/kg	34.5±0.82 ^a^	33.9±0.70 ^a^
Isocoum 10 mg/kg	35.6±0.54 ^a^	34.8±0.66 ^a^
Isocoum 20 mg/kg	34.0±0.54 ^a^	33.0±0.52 ^a^
Cyclophosphamide (CP)	35.2±1.02 ^a^	33.0±1.05 ^a^
CP + Isocoum 5 mg/kg	35.8±0.82 ^a^	34.7±0.60 ^a^
CP + Isocoum 10 mg/kg	35.7±0.63 ^a^	34.6±0.73 ^a^
CP + Isocoum 20 mg/kg	34.1±0.47 ^a^	32.7±0.66 ^a^
Cisplatin (CDDP)	35.5±0.96 ^a^	34.2±0.64 ^a^
CDDP + Isocoum 5 mg/kg	34.3±0.60 ^a^	33.7±0.40 ^a^
CDDP + Isocoum 10mg/kg	34.1±0.68 ^a^	32.1±0.78 ^a^
CDDP + Isocoum 20mg/kg	34.7±0.67 ^a^	33.7±0.89 ^a^

SE – Standard error. Negative control - DMSO 1%; Cyclophosphamide – 100 mg/kg bw., ip.; Cisplatin – 6 mg/kg, bw., ip. Isocoum. – Isocoumarin at doses 5, 10, 20 mg/kg bw, ip. CP + Isocoum. – Cyclophosphamide – 100 mg/kg (p.c.; i.p.) + Isocoumarin at doses 5, 10, 20 mg/kg bw., ip.; CDDP + Isocoum. – Cisplatin – 6mg/kg bw., ip., + Isocoumarin at doses 5, 10, 20 mg/kg bw., ip.; Different letters indicate statistically significant differences (p < 0.05); ANOVA with Tukey post-hoc test.

**Table 2:** Means±SE of the absolute weights of the animals’ organs after the experimentation period.

Experimental groups	Heart	Lungs	Liver	Kidneys	Spleen
Absolute weight (g)					
Negative control	0.202±0.006 ^a^	0.1750.005 ^a^	1.650±0.053 ^a^	0.490±0.015 ^a^	0.1580.007 ^a^
Isocoum 5 mg/kg	0.230±0.009 ^a^	0.311±0.010 ^c^	1.781±0.035 ^a^	0.508±0.022 ^a^	0.230±0.010 ^b^
Isocoum 10 mg/kg	0.232±0.008 ^a^	0.316±0.013 ^c^	1.798±0.029 ^a^	0.500±0.023 ^a^	0.233±0.010 ^b^
Isocoum 20 mg/kg	0.231±0.007 ^a^	0.315±0.009 ^c^	1.780±0.025 ^a^	0.507±0.013 ^a^	0.231±0.012 ^b^
Cyclophosphamide (CP)	0.231±0.006 ^a^	0.2240.006 ^b^	1.663±0.039 ^a^	0.5250.014 ^a^	0.191±0.006 ^ab^
CP + Isocoum 5 mg/kg	0.234±0.006 ^a^	0.260±0.007 ^b^	1.681±0.028 ^a^	0.491±0.014 ^a^	0.1950.016 ^ab^
CP + Isocoum 10 mg/kg	0.235±0.011 ^a^	0.230±0.009 ^b^	1.650±0.059 ^a^	0.490±0.023 ^a^	0.199±0.009 ^ab^
CP + Isocoum 20 mg/kg	0.230±0.013 ^a^	0.230±0.008 ^b^	1.696±0.053 ^a^	0.494±0.008 ^a^	0.197±0.005 ^ab^
Cisplatin (CDDP)	0.234±0.013 ^a^	0.2280.015 ^b^	1.665±0.030 ^a^	0.523±0.016 ^a^	0.193±0.005 ^ab^
CDDP + Isocoum 5 mg/kg	0.213±0.008 ^a^	0.217±0.007 ^ab^	1.693±0.073 ^a^	0.500±0.017 ^a^	0.198±0.008 ^ab^
CDDP + Isocoum 10mg/kg	0.211±0.010 ^a^	0.2180.006 ^ab^	1.699±0.041 ^a^	0.486±0.013 ^a^	0.197±0.009 ^ab^
CDDP + Isocoum 20mg/kg	0.214±0.009 ^a^	0.213±0.033 ^ab^	1.703±0.035 ^a^	0.488±0.016 ^a^	0.198±0.004 ^ab^

SE – Standard error. Negative control - DMSO 1%; Cyclophosphamide – 100 mg/kg bw., ip.; Cisplatin – 6 mg/kg, bw., ip. Isocoum. – Isocoumarin at doses 5, 10, 20 mg/kg bw, ip. CP + Isocoum. – Cyclophosphamide – 100 mg/kg (p.c.; i.p.) + Isocoumarin at doses 5, 10, 20 mg/kg bw., ip.; CDDP + Isocoum. – Cisplatin – 6 mg/kg bw., ip., + Isocoumarin at doses 5, 10, 20 mg/kg bw., ip.; Different letters indicate statistically significant differences (p < 0.05); ANOVA with Tukey post-hoc test.

#### Peripheral blood micronucleus assay

A significant increase in the frequency of micronuclei was observed in the groups treated with the three doses of isocoumarins (5, 10 and 20 mg/kg) at 24 (T1), 48 (T2) and 72 hours (T3) (Table 3). These results indicate that isocoumarin exhibits genotoxic activity. A decrease in micronucleus frequency was observed in the groups treated with isocoumarin in combination with either cyclophosphamide or cisplatin. These results suggest a decrease in the genotoxic effects of both chemotherapeutic agents in combination with isocoumarin.

**Table 3:** Mean frequencies±SE of the damage reduction percentage as determined by the micronucleus assay in mouse peripheral blood cells.

Experimental groups		Mean±SE			DR%	
	24h (T1)	48h (T2)	72h (T3)	24h (T1)	48h (T2)	72h (T3)
Negative control	9.4±0.60^a^	8.4±0.67^a^	8.0±0.51^a^	–	–	–
Cyclophosphamide (CP)	110.4±1.27^g^	97.3±0.37^e^	91.51.08^d^	–	–	–
Isocoum 5 mg/kg	23.4±0.96^c^	16.3±0.84^b^	23.7±1.06^b^	–	–	–
Isocoum 10 mg/kg	33.4±1.22^d^	22.6±0.76^c^	27.8±0.90^b^	–	–	–
Isocoum 20 mg/kg	66.1±1.51^f^	29.1±0.67^d^	37.1±1.31^c^	–	–	–
CP + Isocoum 5 mg/kg	16.5±0.72^b^	23.5±0.80^c^	37.4±1.19^c^	93.0	83.0	64.8
CP + Isocoum 10 mg/kg	33.2±1.14^d^	23.7±0.79^c^	28.4±0.98^b^	76.4	82.8	75.6
CP + Isocoum 20 mg/kg	55.81.25^e^	25.5±0.78^c^	33.7±1.36^c^	54.0	80.7	69.2
Negative control	9.4±0.60^a^	8.4±0.67^a^	7.8±0.49^a^	–	–	–
Cisplatin (CDDP)	93.6±1.90^g^	89.8±1.12^g^	87.9±1.46^e^	–	–	–
Isocoum 5 mg/kg	23.4±0.96^b^	16.3±0.84^b^	23.7±1.06^b^	–	–	–
Isocoum 10 mg/kg	33.4±1.22^c^	22.6±0.76^c^	27.8±0.90^b^	–	–	–
Isocoum 20 mg/kg	66.1±1.51^f^	29.1±0.67^d^	37.1±1.31^c^	–	–	–
CDDP + Isocoum 5 mg/kg	49.7±1.44^e^	57.5±0.97^f^	38.2±1.29^c^	52.1	39.7	62.0
CDDP + Isocoum 10mg/kg	41.0±1.23^d^	30.4±1.54^d^	48.0±1.37^d^	62.5	73.0	49.8
CDDP + Isocoum 20mg/kg	49.5±1.46^e^	38.5±0.99^e^	52.5±1.26^d^	52.4	63.0	44.2

SE – Standard error. Negative control - DMSO 1%; Cyclophosphamide – 100 mg/kg bw., ip.; Cisplatin – 6 mg/kg, bw., ip. Isocoum. – Isocoumarin at doses 5, 10, 20 mg/kg bw, ip. CP + Isocoum. – Cyclophosphamide – 100 mg/kg (p.c.; i.p.) + Isocoumarin at doses 5, 10, 20 mg/kg bw., ip.; CDDP + Isocoum. – Cisplatin – 6 mg/kg bw., ip., + Isocoumarin at doses 5, 10, 20 mg/kg bw., ip.; DR% - percent damage reduction. Different letters indicate statistically significant differences (p < 0.05); ANOVA with Tukey post-hoc test.

#### Cell death assay

The administration of isocoumarin at 5, 10 and 20 mg/kg caused increases (p < 0.05) in the rate of dead cells in the liver of approximately 1.79-, 1.76- and 2.45-fold, respectively, compared to that of the negative control. An increase in the frequency of dead cells was also observed in kidney (p < 0.05) compared to that of the negative control group. Additionally, the rate of dead cells was augmented by the administration of either cyclophosphamide or cisplatin alone (p < 0.05) compared to that of the negative control group. The administration of cyclophosphamide resulted in increases in the frequencies of dead cells of approximately 2.47-fold in the liver and 1.66-fold in the kidney compared to those of the negative control group. The administration of the chemotherapeutic drug cisplatin increased the dead cell rates by 2.09- and 1.76-fold in the liver and kidneys, respectively, compared to those of the negative control group. Combination group II presented a significant increase in the frequency of dead cells in the liver compared to that of the cisplatin group. In the kidneys, the increases in the dead cell rates were approximately 1.05-fold and 1.03-fold with 5 and 20 mg/kg doses, respectively, compared with those of the cisplatin group. Additionally, an increase in the frequency of dead cells was observed in the kidneys of combination group I compared to that of the cyclophosphamide group (Table 4).

**Table 4:** Absolute values (AV) and mean±SE of dead cells in kidneys and liver of Swiss mice.

Experimental groups	Total of cells	Number of dead cells					
		Liver			Kidneys		
		AV	Mean±SE	(%)	AV	Mean±SE	(%)
Negative control	1000	147	14.7±0.56 ^a^	14.7	164	16.4±0.62 ^a^	16.4
Cyclophosphamide (CP)	1000	363	36.3±1.13 ^d^	36.3	273	27.3±0.73 ^b^	27.3
Isocoum 5 mg/kg	1000	264	26.4±1.16 ^b^	26.4	274	27.4±0.65 ^bc^	27.4
Isocoum 10 mg/kg	1000	259	25.9±1.20 ^b^	25.9	260	26.0±0.77 ^b^	26.0
Isocoum 20 mg/kg	1000	361	36.1±0.75 ^d^	36.1	267	26.7±0.73 ^b^	26.7
CP + Isocoum 5 mg/kg	1000	368	36.8±0.70 ^d^	36.8	307	30.7±0.98 ^cd^	30.7
CP + Isocoum 10 mg/kg	1000	310	31.0±0.54 ^c^	31.0	313	31.3±0.77 ^d^	31.3
CP + Isocoum 20 mg/kg	1000	360	36.0±0.54 ^d^	36.0	310	31.0±0.68 ^d^	31.0
Negative control	1000	147	14.7±0.56 ^a^	14.7	164	16.4±0.62 ^a^	16.4
Cisplatin (CDDP)	1000	307	30.7±1.50 ^c^	30.7	289	28.9±0.81 ^bcd^	28.9
Isocoum 5 mg/kg	1000	264	26.4±1.16 ^bc^	26.4	274	27.4±0.65 ^bcd^	27.4
Isocoum 10 mg/kg	1000	259	25.9±1.20 ^b^	25.9	260	26.0±0.77 ^b^	26.0
Isocoum 20 mg/kg	1000	361	36.1±0.75 ^d^	36.1	267	26.7±0.73 ^bc^	26.7
CDDP + Isocoum 5 mg/kg	1000	340	34.0±1.01 ^cd^	34.0	304	30.4±0.76 ^d^	30.4
CDDP + Isocoum 10 mg/kg	1000	334	33.4±1.13 ^cd^	33.4	286	28.6±0.62 ^bcd^	28.6
CDDP + Isocoum 20 mg/kg	1000	350	35.0±1.02 ^cd^	35.0	298	29.8±0.77 ^cd^	29.8

SE – Standard error. Negative control - DMSO 1%; Cyclophosphamide – 100 mg/kg bw., ip.; Cisplatin – 6 mg/kg, bw., ip. Isocoum. – Isocoumarin at doses 5, 10, 20 mg/kg bw, ip. CP + Isocoum. – Cyclophosphamide – 100 mg/kg (p.c.; i.p.) + Isocoumarin at doses 5, 10, 20 mg/kg bw., ip.; CDDP + Isocoum. – Cisplatin – 6 mg/kg bw., ip., + Isocoumarin at doses 5, 10, 20 mg/kg bw., ip.; Different letters indicate statistically significant differences (p < 0.05); ANOVA with Tukey post-hoc test.

#### Splenic phagocytosis

The administration of the new isocoumarin at doses of 5, 10 and 20 mg/kg demonstrated its ability to alter splenic phagocytosis as evidenced by increases in the numbers of cells with phagocytosis (p < 0.05) of approximately 1.69-, 1.71-, and 1.71-fold compared to those of the negative control group. The combination of isocoumarin dosages with cyclophosphamide or cisplatin also increased phagocytic events. An increase in the mean frequency of cells with evidence of splenic phagocytosis was observed in combination group I compared to that in the cyclophosphamide group. The same tendency was observed for combination group II, which also presented an increased frequency of cells with splenic phagocytosis compared to that of the cisplatin group (Table 5).

**Table 5:** Analyzed cell numbers, absolute values (AV), mean frequency±SE, and cell percentages with or without evidence of splenic phagocytosis in Swiss mice treated with different doses of isocoumarin.

Experimental groups	Total of cells	Cells without phagocytosis evidence			Cells with phagocytosis evidence		
		AV	Mean±SE	(%)	AV	Mean±SE	(%)
Negative control	1000	495	49.5±0.40 ^d^	49.5	505	50.5±0.40 ^a^	50.5
Cyclophosphamide (CP)	1000	344	34.4±0.79 ^c^	34.4	656	65.6±0.79 ^b^	65.6
Isocoum 5 mg/kg	1000	144	14.4±0.65 ^a^	14.4	856	85.6±0.65 ^d^	85.6
Isocoum 10 mg/kg	1000	134	13.4±0.40 ^a^	14.0	866	86.6±0.40 ^d^	86.6
Isocoum 20 mg/kg	1000	138	13.8±0.83 ^a^	13.8	862	86.2±0.83 ^d^	86.2
CP + Isocoum 5 mg/kg	1000	261	26.1±0.64 ^b^	26.1	739	73.9±0.64 ^c^	73.9
CP + Isocoum 10 mg/kg	1000	250	25.0±0.39 ^b^	25.0	750	75.0±0.39 ^c^	75.0
CP + Isocoum 20 mg/kg	1000	260	26.0±0.76 ^b^	26.0	740	74.0±0.76 ^c^	74.0
Negative control	1000	495	49.5±0.40 ^d^	49.5	505	50.5±0.40 ^a^	50.5
Cisplatin (CDDP)	1000	403	40.3±0.42 ^c^	40.3	597	59.7±0.42 ^b^	59.7
Isocoum 5 mg/kg	1000	144	14.4±0.66 ^a^	14.4	856	85.2±0.66 ^d^	85.2
Isocoum 10 mg/kg	1000	134	13.4±0.40 ^a^	13.4	866	86.6±0.40 ^d^	86.6
Isocoum 20 mg/kg	1000	138	13.8±0.83 ^a^	13.8	862	86.2±0.83 ^d^	86.2
CDDP + Isocoum 5 mg/kg	1000	361	36.1±0.64 ^b^	36.2	639	63.9±0.64 ^c^	63.9
CDDP + Isocoum 10 mg/kg	1000	366	36.6±0.75 ^b^	36.6	634	63.4±0.75 ^c^	63.4
CDDP + Isocoum 20 mg/kg	1000	358	35.8±0.88 ^b^	35.8	642	64.2±0.88 ^c^	64.2

SE – Standard error. Negative control - DMSO 1%; Cyclophosphamide – 100 mg/kg bw., ip.; Cisplatin – 6 mg/kg, bw., ip. Isocoum. – Isocoumarin at doses 5, 10, 20 mg/kg bw, ip. CP + Isocoum. – Cyclophosphamide – 100 mg/kg (p.c.; i.p.) + Isocoumarin at doses 5, 10, 20 mg/kg bw., ip.; CDDP + Isocoum. – Cisplatin – 6 mg/kg bw., ip., + Isocoumarin at doses 5, 10, 20 mg/kg bw., ip.; Different letters indicate statistically significant differences (p < 0.05); ANOVA with Tukey post-hoc test.

#### Differential blood cell count

The differential counting of blood cells showed that the administration of the new isocoumarin at doses of 5, 10 and 20 mg/kg increased lymphocyte numbers and reduced neutrophil number (p < 0.05). The isocoumarin, cyclophosphamide and cisplatin groups presented higher mean numbers of monocytes compared to the reference values. Moreover, an increase in the mean number of monocytes was observed in the isocoumarin group compared to that in the negative control group (Table 6).

**Table 6:** Reference values and mean±SE of the differential blood cell count in Swiss mice treated with different doses of isocoumarin.

Cell type	Lymphocytes	Neutrophils	Monocytes	Eosinophils	Basophils
Reference values	55-95%	10-40%	0.1-3.5%	0-0.4%	0-0.3%
Experimental groups					
Negative control	49.2±2.10 ^a^	45.8±1.62 ^e^	3.2±0.42 ^abc^	1.8±0.63 ^ab^	0.0±0.00 ^a^
Cyclophosphamide (CP)	54.2±0.79 ^b^	39.7±0.82 ^d^	5.2±0.42 ^d^	0.9±057 ^a^	0.0±0.00 ^a^
Isocoum 5 mg/kg	52.2±1.81 ^ab^	33.71.25 ^c^	4.2±0.42 ^cd^	9.9±0.99 ^b^	0.0±0.00 ^a^
Isocoum 10 mg/kg	62.3±2.00 ^c^	28.0±1.33 ^b^	4.5±0.71 ^cd^	5.2±0.63 ^b^	0.0±0.00 ^a^
Isocoum 20 mg/kg	67.8±1.47 ^d^	27.0±2.11 ^b^	3.9±0.74 ^bcd^	1.3±1.16 ^a^	0.0±0.00 ^a^
CP + Isocoum 5 mg/kg	76.5±6.96 ^e^	21.4±6.52 ^a^	1.4±0.51 ^a^	0.7±0.48 ^a^	0.0±0.00 ^a^
CP + Isocoum 10 mg/kg	68.5±1.08 ^d^	28.2±1.13 ^b^	2.2±0.79 ^ab^	1.1±0.31 ^a^	0.0±0.00 ^a^
CP + Isocoum 20 mg/kg	79.3±1.83 ^e^	17.9±1.85 ^a^	1.8±0.63 ^a^	1.0±0.00 ^a^	0.0±0.00 ^a^
Negative control	49.2±2.10 ^a^	45.8±1.62 ^e^	3.2±0.42 ^bcd^	1.8±0.63 ^ab^	0.0±0.00 ^a^
Cisplatin (CDDP)	56.3±2.16 ^c^	37.7±2.83 ^d^	4.6±1.26 ^d^	1.50.71 ^a^	0.0±0.00 ^a^
Isocoum 5 mg/kg	52.2±1.81 ^b^	33.71.25 ^c^	4.2±0.42 ^d^	9.9±0.99 ^b^	0.0±0.00 ^a^
Isocoum 10 mg/kg	62.3±2.00 ^d^	28.0±1.33 ^b^	4.5±0.71 ^d^	5.2±0.63 ^b^	0.0±0.00 ^a^
Isocoum 20 mg/kg	67.8±1.47 ^e^	27.0±2.11 ^b^	3.9±0.74 ^cd^	1.3±1.16 ^a^	0.0±0.00 ^a^
CDDP + Isocoum 5 mg/kg	81.0±0.81 ^f^	16.8±0.92 ^a^	2.1±0.57 ^abc^	0.1±0.31 ^a^	0.0±0.00 ^a^
CDDP + Isocoum 10 mg/kg	66.2±2.04 ^e^	33.2±1.87 ^c^	0.1±0.31 ^a^	0.5±0.53 ^a^	0.0±0.00 ^a^
CDDP + Isocoum 20 mg/kg	80.7±1.50 ^f^	18.61.58 ^a^	0.6±0.51 ^ab^	0.1±0.31 ^a^	0.0±0.00 ^a^

SE – Standard error. Negative control - DMSO 1%; Cyclophosphamide – 100 mg/kg bw., ip.; Cisplatin – 6 mg/kg, bw., ip. Isocoum. – Isocoumarin at doses 5, 10, 20 mg/kg bw, ip. CP + Isocoum. – Cyclophosphamide – 100mg/kg (p.c.; i.p.) + Isocoumarin at doses 5, 10, 20 mg/kg bw., ip.; CDDP + Isocoum. – Cisplatin – 6 mg/kg bw., ip., + Isocoumarin at doses 5, 10, 20 mg/kg bw., ip.; Different letters indicate statistically significant differences (p < 0.05); ANOVA with Tukey post-hoc test.

## Discussion

The search for new, pharmacologically functional, phenolic compounds has led to the discovery and synthesis of many drugs with anticancer activity (Queiroz *et al.*, 2009; Kaminskyy *et al.*, 2014; Navarro *et al.*, 2014). These compounds include synthetic isocoumarins (Yin *et al.*, 2001), which present antioxidant, antitumor and anti-inflammatory activity *in vivo* (Yin *et al.*, 2001; Curini *et al.*, 2004; Lacy and O’Kennedy, 2004).

Genotoxicity, immunomodulation and apoptosis assays are suitable for the toxicological assessment of new drug candidates (Lacy and O’Kennedy, 2004) and were used in this study (Comet/micronucleus – genotoxicity; alteration of spleen phagocytosis and leukometry suggesting immunomodulation; and the cell death assay). Also, these tests are considered fundamental pre-indicative models for evaluating the frequencies of DNA and chromosome damage that are able to induce cancer development (Lacy and O’Kennedy, 2004; Mauro *et al.*, 2011).

The new isocoumarin demonstrated high genotoxic potential in a micronucleus test. The toxic effects of many coumarins have been studied by the Food and Drug Administration (FDA) and by the United States National Toxicology Program of the National Institute of Environmental Health Sciences due to the use of such compounds on a large scale in the production of fragrances and flavor enhancers (Yourick and Bronaugh, 1997). High doses of coumarin are toxic and have genotoxic potential in *in vivo* experiments with rats and mice (Lake, 1984, 1999; Yin *et al.*, 2001); however, doses that are toxic in rats are not toxic for humans (Lacy and O’Kennedy, 2004; Napimogaa and Yatsuda, 2010). The new isocoumarin synthesized in this study is classified as a phenolic lipid because a hydrophobic chain was incorporated into the initial coumarin (compound 2). Phenolic lipids are known to have antigenotoxic potential (Parikka *et al.*, 2006; Melo-Cavalcante *et al.*, 2008; Stasiuk and Kozubek, 2010; Navarro *et al.*, 2014). However, the new isocoumarin exhibited strong genotoxic activity, which is consistent with the known high genotoxic potential resulting from the chemical properties of coumarins (Lake, 1984, 1999; Yourick and Bronaugh, 1997; Born *et al.*, 1999; Borges *et al.*, 2009; Napimogaa and Yatsuda, 2010).

The new isocoumarin presents a hydrophobic chain, which is likely related to DNA protection, and a phenolic ring, which is likely related to DNA damage. Thus, these two chemical characteristics are incorporated in the same compound, and an antagonist activity is observed. When tested alone, the new isocoumarin has genotoxic potential, and when tested in combination with either chemotherapeutic agent (cisplatin or cyclophosphamide), antigenotoxic and chemopreventive activities were observed. Consequently, we hypothesize that this new compound has bifunctional activity.

Our results corroborate results obtained by other authors regarding antigenotoxic activity (Melo-Cavalcante *et al.*, 2008; Stasiuk and Kozubek, 2010). The new isocoumarin presented a substantial DR%, which is indicative of great chemoprevention when combined with cyclophosphamide or cisplatin. Additionally, the results did not show a dose-response relationship or a correlation between the time of compound metabolism and DNA damage reduction.

The various classes of coumarin have been widely studied for their antioxidant activities (Nishiyama *et al.*, 2001; Di Stasi *et al.*, 2004; Fylaktakidou, *et al.*, 2004; Luchini *et al.*, 2008). Antioxidant characteristics are associated with anti-inflammatory/inflammatory (Fedel-Miyasato *et al.*, 2014), immunomodulatory (Murakami *et al.*, 2000; Ishii *et al.*, 2011) and chemopreventive activity (Oliveira *et al.*, 2013, 2014). Superoxide anions are free radicals that can cause lesions in the DNA and in tissues, primarily because of the infiltration of neutrophils in the tissues and because of the action of the phagocytary neutrophils (Murakami *et al.*, 2000). According to Murakami *et al.* (2000), 7,8-dihydroxy coumarins are capable of sequestering superoxide anions and thus of exerting antioxidative action. Considering these findings, this sequestering could be responsible for the chemopreventive potential (Oliveira *et al.*, 2006) of this new isocoumarin.

The anti-cancer activity of coumarin was confirmed in a study that used a compound extracted from fresh leaves of *Mikania glomerata* (Napimogaa and Yatsuda, 2010). Other studies have described synthetic and natural coumarins as promising compounds for the treatment of cancer because of their toxic properties resulting from their benzene and 1,2-pyrone rings (Lake, 1984, 1999; Born *et al.*, 1999; Yin *et al.*, 2001; Lacy and O’Kennedy, 2004; Chen *et al.*, 2007; Borges *et al.*, 2009; Napimogaa and Yatsuda, 2010). The present research provided an initial analysis of this new isocoumarin, and great genotoxic activity was observed. Such potential could classify this new compound as a chemotherapeutic agent. The frequencies of damage observed for this new isocoumarin are 59.9 and 70.6% of the damage caused by cyclophosphamide and cisplatin, respectively. However, when the new isocoumarin was combined with either chemotherapeutic agents, antigenotoxic activity was observed, which could compromise the capacity of DNA damage induction, thus compromising apoptosis caused by chemotherapy. This fact eliminates the possibilities of the combined administration of the new isocoumarin with these chemotherapeutic agents. Additionally, our results are in disagreement with data observed in the study by Navarro *et al.* (2014) because these authors found that the resorcinolic lipid AMS35AA has the capacity to potentiate the chemotherapeutic effect of cyclophosphamide. We infer from these discrepancies that different phenolic lipids have different modes of action.

Regarding dead cells in the liver and in the kidneys, the new isocoumarin promoted the same frequencies as did cyclophosphamide and cisplatin. In addition, the strong genotoxic potential of the new isocoumarin suggests that further studies are needed to understand and verify its chemotherapy potential. The toxic, apoptotic, and genotoxic potential of coumarins has already been described by Yin *et al.* (2001); Agata *et al.* (2004); Chen *et al.* (2007).

Importantly, coumarins are capable of increasing apoptotic events in tissues through their benzene and 1,2-pyrone rings. Although the mechanisms of coumarin-induced apoptosis are not entirely clear, different studies show that coumarins may increase the frequency of apoptosis by modulating pro-apoptotic genes (Bhattacharyya *et al.*, 2009; Zhang *et al.*, 2012; Shokoohinia *et al.*, 2014). Thus, the mechanism of cell death would not involve DNA damage. Therefore, the new isocoumarin herein tested could exert both chemopreventive potential and induce cell death by apoptosis.

When combining the new isocoumarin with either chemotherapeutic agent, the inhibition and induction of apoptosis were not observed. Because a chemopreventive potential was observed, as shown by the high DR% in the combination groups, a reduction in apoptosis was expected, but it did not occur. Thus, further studies of this new compound are required. Vukicevic *et al.* (2004) and Oliveira *et al.* (2007) have already shown that the frequency of apoptotic cells can be maintained when facing genetic damage that did not develop into micronuclei. Thus, the frequency of apoptotic events caused by an alkylating agent can be maintained even in the presence of chemopreventive compounds.

When analyzing the results of the splenic phagocytosis assay, we observed that both cyclophosphamide and cisplatin promoted increases in the frequency of micronuclei and, thus, increases in splenic phagocytosis. Splenic phagocytosis is a mechanism used to remove injured cells from the body, including those with micronuclei (Ishii *et al.*, 2011; Fedel-Miyasato *et al.*, 2014). When the new isocoumarin was administered alone, an increase in splenic phagocytosis was observed. The ability to alter splenic activity and chemotherapy potential were observed for the isocoumarin highest tested dose. Additionally, the increases in apoptotic events in the kidneys and liver corroborate these findings because they are significant when compared to the capacities of both positive controls (cyclophosphamide and cisplatin) to induce apoptosis in these organs.

Phagocytic activity also increased when the new isocoumarin was administered in combination with either chemotherapy drug. However, this particular increase was lower than that observed with the new isocoumarin administered alone. When interpreting this result, three events must be considered: the activity of chemoprevention, the maintenance of apoptotic events and the increase in phagocytic events. Generally, antimutagenesis modes of action are described as desmutagenesis and bioantimutagenesis (Oliveira *et al.*, 2007, 2009a, 2009b, 2013, 2014). Therefore, antimutagenesis also occurs by splenic activity (Ishii *et al.*, 2011; Fedel-Miyasato *et al.*, 2014; Navarro *et al.*, 2014). Thus, the chemopreventive activity of the new isocoumarin could be related to the increase in splenic phagocytosis, in addition to desmutagenesis and bioantimutagenesis, which are classic modes of action described by by Kada *et al.* (1982) and Kada and Shimoi (1987) and are now considered also genotoxic damage. If cells with micronuclei (genotoxic lesions) are removed from the blood by splenic phagocytosis before apoptosis occurs, then maintaining the frequency of apoptosis in the liver and kidneys can be explained even in the presence of a chemopreventive effect. Taken together, these data suggest that apoptosis could be responsible for the removal of damaged cells from the body, preventing their identification and quantification in peripheral blood by the micronucleus assay (Vukicevic *et al.*, 2004; Oliveira *et al.*, 2007).

The differential blood cell count supports the immunomodulatory activity that was observed in the phagocytosis assay. The reduction of neutrophils is related to the increase in splenic phagocytosis because of the migration of neutrophils to tissues (*e.g.*, the spleen) where they exert their phagocytic function (Luchini *et al.*, 2008). Additionally, the new isocoumarin, alone or in combination with either chemotherapeutic agent, is capable of increasing the frequency of lymphocytes. Patients who undergo chemotherapy become immunosuppressed (Rasmussen and Arvin, 1982), and reduced frequencies of lymphocytes compromise new cycles of chemotherapy. This important adverse effect is undesirable in new chemotherapy drugs (Navarro *et al.*, 2014).

The new isocoumarin exhibits more than one characteristic of chemotherapeutic agents. Although it promoted an increase in splenic activity and altered the leukometry in animals treated with cyclophosphamide and cisplatin, the cost *vs.* benefit analysis of this drug must be evaluated because the combination of these compounds reduced the frequency of genotoxic damage and maintained apoptotic events, thus reducing the chemotherapeutic effects of cisplatin and cyclophosphamide. Therefore, the capability of the new isocoumarin to increase splenic activity and alter leukometry when administered alone, as well as its genotoxic and apoptotic activities, and phagocytosis induction potential are important characteristics in the search for a new chemotherapy agent and must be considered in the development of new antitumor compounds.

Although the new isocoumarin is not likely to be an adjuvant to chemotherapy treatments that use cyclophosphamide or cisplatin, the compound merits to be considered as a prototype for the development of new antitumor drugs.
